# Dietary sodium intake and overweight and obesity in children and adults: a protocol for a systematic review and meta-analysis

**DOI:** 10.1186/s13643-015-0175-3

**Published:** 2016-01-18

**Authors:** Carley A. Grimes, Dieuwerke P. Bolhuis, Feng J. He, Caryl A. Nowson

**Affiliations:** Centre for Physical Activity and Nutrition Research, School of Exercise and Nutrition Sciences, Deakin University, 221 Burwood Highway, Burwood, Victoria 3125 Australia; Centre for Advanced Sensory Science, School of Exercise and Nutrition Sciences, Deakin University, 221 Burwood Highway, Burwood, Victoria 3125 Australia; Centre for Environmental and Preventative Medicine, Wolfson Institute of Preventative Medicine, Queen Mary University of London, Charterhouse Square, London, EC1M 6BQ UK; Centre for Physical Activity and Nutrition Research, School of Exercise and Nutrition Sciences, Deakin University, 75 Pigdons Road, Waurn Ponds Campus, Geelong, Victoria 3216 Australia

**Keywords:** Dietary salt, Dietary sodium, Sodium chloride, Adiposity, Obesity, Body mass index, Body weight, Sugar-sweetened beverage

## Abstract

**Background:**

Overweight and obesity in children and adults is a major public health concern. Emerging evidence suggests dietary sodium intake may be associated with obesity. This systematic review and meta-analysis will aim to (i) assess the relation between dietary sodium intake and measures of adiposity in children and adults and (ii) examine the relation between sodium intake and sugar-sweetened beverage (SSB) consumption, which is a known risk factor for obesity.

**Methods/design:**

An electronic search will be conducted using Medline Complete, CINAHL, Scopus, Embase and Cochrane central register of controlled trials (CENTRAL). The search strategy will identify published peer-reviewed articles that report on dietary sodium and either a marker of adiposity or SSB consumption. Only human studies (ages >1 year) in English will be included, and no limits will be placed on publication date. No restrictions will be placed on the method of sodium intake assessment. Cross-sectional, prospective studies, and randomised controlled trials with a duration of ≥3 months will be included. Studies with participants with renal disease, cancer, type 1 diabetes or heart failure or who are pregnant will be excluded. To assess the quality of studies, the Cochrane’s Collaboration tool for assessing risk of bias in randomised trials will be used for randomised controlled trials (RCTs), and the modified Newcastle-Ottawa Scale will be used for cross-sectional and prospective studies. Meta-analysis will be used to assess the relation of sodium intake with two primary outcomes: (i) BMI and body weight in adults and BMI z-score in children and (ii) weight category (i.e. healthy weight vs. overweight/obese). For any outcomes in which meta-analysis is not possible, we will present data as a systematic review. Findings will be grouped and reported separately for children and adolescents (ages 1–17 years) and adults (ages >18 years).

**Discussion:**

This review and meta-analysis will provide insight into the relation between dietary sodium intake and overweight and obesity. This information can be used to inform public health policies which target population sodium consumption.

**Systematic review registration:**

Prospero CRD42015016440

**Electronic supplementary material:**

The online version of this article (doi:10.1186/s13643-015-0175-3) contains supplementary material, which is available to authorized users.

## Background

In many countries, overweight and obesity in children and adults is a major public health issue [1]. The consequences of obesity are substantial and include adverse health and psychological outcomes and increased economic burden [2]. The accumulation of excess weight during childhood is particularly concerning as it promotes the early onset of chronic diseases, such as cardiovascular disease and diabetes [3], and increases the risk of being overweight and obese in adulthood [4]. Whilst the causes of obesity are complex and multifactorial, an overall unhealthy diet characterised by excess energy is considered to be the cornerstone for excess weight gain [5, 6].

Recently, a number of studies have emerged which suggest that dietary sodium intake may be implicated in weight gain. Studies in children [7–9] and adults [8, 10–12] have reported positive associations between sodium intake and a range of adiposity outcomes including BMI or in the case of children BMI z-score, weight category, percent body fat and abdominal obesity. Cross-sectional studies conducted in children from the UK, USA and Australia have shown dietary sodium intake is positively associated with the consumption of energy-rich sugar-sweetened beverages [13–15]. It has been postulated that this relationship may be due to the effects of sodium on thirst, as experimental studies in both animals and humans show increased fluid intake on a higher sodium diet [16, 17].

Reported associations between sodium intake and adiposity outcomes may be confounded by energy intake, as foods high in sodium are often also high in energy. However, it may also be the case that energy intake is a mediating factor on the causal pathway between sodium intake and obesity. The addition of sodium chloride (salt) increases the palatability of many foods and encourages greater energy intake [18]. Moreover, it has been suggested that salt may act as a vehicle that drives intake of dietary fat. This is supported by reports which show that attraction to salty-and-fatty foods are associated with higher total daily energy intakes in adults [19], uncontrolled eating [20] and overweight in children [21].

Interestingly, some studies report an association between sodium intake and adiposity measures, which are independent of energy intake [7–9]. In humans, an alternative mechanism which may explain this relationship remains unclear. However, findings from animal studies indicate that a diet high in sodium increases adipose tissue mass, and this is due to changes in insulin and glucose metabolism which favour fat accumulation [22, 23]. For example, in a study of male Wister rats, it was shown that those rats who were fed a high sodium diet had significant increases in adipose tissue mass from all three sites of collection (i.e. subcutaneous, periepididymal and retroperitoneal fat pads) at week 6 compared to those rats fed a normal sodium diet; this was despite no difference in energy intakes between the groups. However, by completion of the intervention (i.e. week 9), there was no difference in adipose tissue mass between the groups [22].

Given the ubiquity of sodium in the food supply [24], it is important to understand if there are additional health concerns of a high sodium diet, which go beyond the traditional concerns of blood pressure and cardiovascular health [25]. To date, the emerging literature surrounding dietary sodium intake and obesity has not been systematically reviewed. This information can be used to inform public health policy which relates to population sodium reduction strategies.

### Objective

The primary aim of this systematic review and meta-analysis is to examine the relation between dietary sodium intake and measures of adiposity in children and adults. The primary outcomes are (i) body weight and BMI for adults and BMI z-score for children and adolescents and (ii) weight category (i.e. ‘healthy weight’ vs. ‘overweight/obese’). Secondary outcomes for adiposity measures include percent body fat and central obesity. A secondary aim is to examine the relation between sodium intake and sugar-sweetened beverage consumption, which is a known risk factor for obesity.

## Methods/design

This protocol adheres to the Preferred Reporting Items for Systematic Review and Meta-Analysis Protocols (PRISMA-P) 2015 statement [26] (Additional file 1) and was registered with the International Prospective Register of Systematic Reviews (PROSPERO) (registration number CRD42015016440).

### Search strategy

An electronic literature search will be conducted using four databases: Medline Complete (EBSCO Host), CINAHL (EBSCO Host), Scopus, Embase and Cochrane central register of controlled trials (CENTRAL). The search strategy was developed in consultation with a research librarian. Free text keywords were used to conduct the search. Medical subject headings (MeSH) were considered in the development of the search terms. E.g. the MeSH term ‘sodium chloride, dietary’ was included as a keyword, therefore within the database Medline Complete articles which include this term as a MeSH Heading would be retrieved (Table 1). Search criteria specific to each database are outlined in Table 2. The search strategy was piloted across each database to improve the effectiveness of the final search. Only peer-reviewed original research articles published in English and conducted in humans will be included. It is beyond the scope of this review to include and examine sources from ‘grey’ literature. The risk of bias and threat to validity by excluding unpublished studies will be discussed within the final manuscript. The reference lists of included studies identified through the search will also be reviewed. Near the end of the review process, the search will be rerun to identify any potential studies that have been published since the initial search.

**Table 1:** Search strategy

Search concept	Search terms
Concept 1 (exposure)	“sodium intake”
	OR “sodium consumption”
	OR “dietary sodium”
	OR “sodium chloride intake”
	OR “sodium chloride consumption”
	OR “dietary sodium chloride”
	OR “salt intake”
	OR “salt consumption”
	OR “dietary salt”
	OR “sodium, dietary” (MeSH Heading)
	OR “salt, dietary”
	OR “sodium chloride, dietary” (MeSH Heading)
Concept 2 (outcome)	“body weight” (MeSH Heading)
	OR bmi
	OR “body mass index” (MeSH Heading)
	OR “bmi score”
	OR “bmi z-score”
	OR “bmi sds”
	OR “body fat” (MeSH Heading)
	OR “body fat percentage”
	OR “% body fat”
	OR “percent body fat”
	OR “fat mass”
	OR “bmi percentile”
	OR obes*
	OR overweight (MeSH Heading)
	OR adipos*
	OR “waist circumference” (MeSH Heading)
	OR “waist circumference z-score”
	OR “waist-to-height-ratio” (MeSH Heading)
	OR “WHtR”
	OR “centrally obese”
	OR “central adiposity”
	OR “body composition” (MeSH Heading)
	OR “sugar-sweetened beverage”
	OR “sugar-sweetened beverages”
Final search	Concept 1 AND Concept 2

In Medline Complete, free text terms were also searched for

**Table 2:** Search criteria specifications for each database

Database	Search options
Medline Complete^a^ via EBSCOhost Research Databases	Limiters: English language, human
	Search mode: Boolean/phrase
CINAHL via EBSCOhost Research Databases	Limiters: English language, human, peer reviewed
	Search mode: Boolean/phrase
Scopus	Limiters: English language; document type: article
	Search mode: Boolean/phrase
Embase	Advanced search
	No mapping options used
	No date limits specified
	Sources: Embase only (Medline not selected as separate search)
	Field labels: abstract, article title, index term and subheading
	Quick limits: human, only in English
	Publication types: article, article in press
	EBM, gender, age and animal advanced options left blank
Cochrane Library	Search: title, abstract, keywords
	Limits: Cochrane central register of controlled trials (CENTRAL)

^a^Medline Complete only includes peer-reviewed journals

### Eligibility criteria

Studies will be included if they report sodium intake and either a measure of an adiposity outcome or sugar-sweetened beverage (SSB) intake. Whilst the primary outcome of this review is a marker of adiposity, we have included SSB intake as a secondary outcome, as early work identified SSBs as a potential mediating factor linking sodium intake to obesity [15, 27]. SSBs will include sugar-sweetened soda, vitamin waters, fruit ades, fruit drinks, squash (i.e. cordial), flavoured mineral waters and sports and energy drinks [28]. Studies that include 100 % fruit juice or sweetened tea or coffee within their definition of SSB will be excluded.

The exposure outcome is dietary sodium intake, and as 90 % of sodium is consumed in the form of sodium chloride, the terms dietary salt and sodium chloride will also be considered as exposure outcomes. Studies which assess sodium intake via dietary method or via urinary electrolyte analyses will be included. No restrictions will be placed on the method of sodium intake assessment (i.e. inclusion of dietary methods: dietary recalls, diet records and food frequency questionnaires; urinary electrolyte assessment: 24-h urine, overnight, timed or spot urine collections). Cross-sectional studies, prospective studies (with at least 1 year duration) and randomised controlled trials (RCTs) will be included. Due to the short-term effects of sodium reduction on extracellular fluid loss and change in body weight, RCTs where the salt reduction arm within the intervention has a duration of less than 3 months will be excluded. Weight loss trials and weight maintenance studies, which are designed to achieve weight loss, will be excluded as it would not be possible to discern the effects of sodium alone (i.e. a range of diet and/or lifestyle factors are generally targeted). In the case of salt reduction, RCTs where there is more than one intervention arm under investigation, for example a weight loss group, sodium reduction only group and combined treatment group (i.e. weight loss and sodium reduction), we will only extract data for the sodium reduction group and control group (i.e. ‘usual care’) and exclude data from the weight loss group. No restrictions will be placed on participants who are taking antihypertensive medications within a salt reduction intervention; however, information related to diuretic use will be recorded in the data extraction form. RCTs that specifically test the effects of diuretic therapy will be excluded. Case control studies will be excluded. Infants, defined as <1 year of age will be excluded, and no other age restrictions will be applied. Participants with renal disease, cancer, type 1 diabetes or heart failure or who are pregnant will be excluded.

### Study selection and data management

All papers identified from the initial electronic search process will be imported into an endnote library, and duplicates will be removed. Titles and abstracts will be screened by two investigators. Studies will be included based on the eligibility criteria as outlined above, and discrepancies in opinion of studies to include at this stage amongst the two reviewers will be resolved through consultation with a third reviewer (CN). Following this screening process, the full text of potential studies to include will be retrieved. Two independent reviewers will screen at the full text stage according to the eligibility criteria. Any discrepancies between the two reviewers for included or excluded studies will be discussed, and if an agreement cannot be reached, a third reviewer (CN) will be used to reach consensus. The reason for excluding each study will be recorded. At this stage, the reference lists of included studies will be scanned, and if any relevant studies are identified, the full text will be retrieved and reviewed for inclusion by both reviewers. Data extraction of included studies will be completed by two independent reviewers (CG, DB) using a data extraction template. The template will include the following: author; title; journal; year of publication; study setting; study design; study population; sample size; participant demographic characteristics; method used to assess sodium intake; intervention details (where applicable), in children methods used to calculate BMI z-score and define overweight and obesity (e.g. CDC vs. WHO BMI cut-offs); study outcomes relevant to the aims of this review, i.e. sodium; and outcomes for adiposity measures or SSB consumption, statistical analysis and confounder adjustment; and funding source. The template will be piloted by both reviewers, and if necessary, the information recorded will be modified. Any disagreement between extracted data between each reviewer (CG, DB) will be discussed, and if an agreement cannot be reached, a third reviewer (CN) will provide input.

### Quality assessment

The quality of studies included in this review will be assessed by two researchers (CG and DB) using a tool appropriate for the study design. Any discrepancies between the two reviewers will be discussed, and if a consensus on study quality rating cannot be reached, advice will be sought from a third reviewer (CN). For RCTs, the Cochrane’s Collaboration tool for assessing risk of bias in randomised trials [29] will be used. This tool includes six domains to assess bias (i.e. selection bias, performance bias, detection bias, attrition bias and reporting bias) which are assigned as either ‘low risk of bias’, ‘unclear risk of bias’ or ‘high risk of bias’ [29]. This information will be presented as a risk of bias summary figure using the Review Manager software (RevMan, version 5.3). To assess the study quality of prospective and cross-sectional studies, a modified version of the Newcastle-Ottawa Scale (NOS) for cohort studies will be used [30] (Additional file 2). The tool has been modified to suit the context of studies that will be included in this review, for example consideration is given to the methodology used to determine sodium intake. This tool assigns stars to indicate higher quality based on three broad criteria, specific to the study design (i.e. selection of study groups, comparability and outcome assessment). This information will be presented in a summary table, indicating the star rating for each individual study included in the review. This process of quality assessment will be completed by two independent reviewers (CG, DB) for each study included in the review.

### Data synthesis

The PRISMA flow chart [31] will be used to document the number of studies identified during the search process and those excluded and included according to the outlined eligibility criteria. Random effects meta-analysis methods will be used to assess the relation between sodium intake and the primary outcomes: (i) body weight and BMI for adults and BMI z-score for children/adolescents and (ii) weight category (i.e. ‘healthy weight’, ‘overweight/obese’). All meta-analyses will be conducted separately for (i) adults (ages ≥18 years) and children and adolescents (ages 1–17 years) and (ii) by study design (i.e. RCTs, prospective and cross-sectional studies). We anticipate that there will be a sufficient number of cross-sectional studies to conduct a meta-analysis for sodium intake and the primary outcomes; however, the decision to complete a meta-analysis of RCTs and prospective studies will be dependent on the number of these types of studies identified in the review. To conduct a meta-analysis, a minimum of two studies will be required. The meta-analytical approach will be dependent on the study design and is summarised below.

RCTs: In adults, the primary outcome will be body weight, rather than BMI. This is because height remains constant in pre- and post-studies and hence does not need to be controlled for. In the case of RCTs in children, due to growth changes, BMI z-score will remain as the primary outcome. Meta-analysis will be used to calculate the weighted mean difference (95 % CI) of change in body weight/BMI z-score between reduced salt and control group intervention arms. Where measures are available at numerous time points, we will use data from the latest time point available.

Prospective and cross-sectional studies: The primary outcomes that will be assessed include BMI in adults, BMI z-score in children and weight category for both adults and children. For continuous outcomes (e.g. BMI and BMI z-score), the pooled β coefficient (95 % CI) for the association between salt intake and BMI/BMI z-score will be reported. For dichotomous outcomes, the pooled odds ratio (95 % CI) for the association between salt intake and weight category (i.e. healthy weight vs. overweight/obese) will be reported. Potential confounders (e.g. age, sex, socio-economic status) will be accounted for by using data from the most fully adjusted model (e.g. covariates such as age, sex, socio-economic status) available. Additional meta-analyses related to sodium intake and other measures of adiposity (e.g. % body fat and central obesity) will only be conducted if a sufficient number of studies which report these outcomes are identified. With reference to the secondary outcome, we anticipate that in children and adolescents there will be enough cross-sectional studies to conduct a meta-analysis to produce a pooled β coefficient (95 % CI) estimate for the association between sodium intake and SSB intake. Based on current work in this area, it is anticipated that the level of sodium exposure will be the equivalent of 1 g of salt per day and its association with SSB intake (g/d). All data from the meta-analyses will be displayed in forest plots. To assess heterogeneity, we will use the *Q* statistic and *I*^2^ index. For the *Q* statistic, a *P* value of <0.10 will be used as a cut-point to indicate heterogeneity; however, consideration will be given to the power of this test if only a few studies and/or studies of small sample size are included in the meta-analysis [32]. The degree of heterogeneity will be assessed using the *I*^2^ index, and the following values will be indicative of moderate (30–50 %), substantial (50–75 %) and considerable heterogeneity (75–100 %) [32]. Potential heterogeneity will be explored by sub-group analysis, for example method of assessing salt intake, adjustment for confounders and duration of intervention. Funnel plots and Egger’s regression test will be used to assess publication bias. All statistical analyses will be conducted using STATA version 14 (Stata Corporation, College Park, TX, USA). The Grading of Recommendations, Assessment, Development and Evaluation (GRADE) framework will be applied to determine an overall evidence rating for the two primary outcomes (BMI/BMI z-score and weight category) included in the meta-analyses [33]. This will be completed separately for adults and children/adolescents. For any outcomes in which meta-analysis is not possible, we will present data as a systematic review. These findings will be presented in summary tables according to the data extraction headings as specified under the data management section. Findings will be grouped and reported separately for children and adolescents (ages 1–17 years) and adults (ages ≥18 years).

## Discussion

This will be the first systematic review and meta-analysis of studies to examine the association between dietary sodium intake and overweight and obesity, as well as sodium and sugar-sweetened beverage intake. Whilst it is already recognised that a diet high in sodium increases cardiovascular risk via the effects of raised blood pressure [25], a greater understanding of any additional cardiovascular risk due to adiposity is important for the direction of public health policy that aims to lower population sodium intake.

## Additional files

Additional file 1:
**PRISMA-P 2015 checklist.** (PDF 110 kb) Additional file 2:
**Modified Newcastle–Ottawa Quality Assessment Scale.** (PDF 272 kb)

## Abbreviations

BMI: body mass index
RCT: randomised controlled trial (body mass index standard deviation score)
SSB: sugar-sweetened beverage
