# Supplementary material for: Dietary sodium intake and overweight and obesity in children and adults: a protocol for a systematic review and meta-analysis
Source: Syst Rev. 2016 Jan 18;5:7. doi: 10.1186/s13643-015-0175-3 (PMC4717573; doi:10.1186/s13643-015-0175-3)
Supplement: Additional file 1: — PRISMA-P 2015 checklist. (PDF 110 kb) [file 13643_2015_175_MOESM1_ESM.pdf]

Table 3 PRISMA-P 2015 checklist: recommended items to include in a systematic review protocol[a]

	Section/topic	Item #	Checklist item
	**ADMINISTRATIVE INFORMATION**		
	**Title**		
P1	**Identification**	1a	Identify the report as a protocol of a systematic review
n/a	**Update**	1b	If the protocol is for an update of a previous systematic review, identify as such
p3	**Registration**	2	If registered, provide the name of the registry (e.g., PROSPERO) and registration number
	**Authors**		
p1	**Contact**	3a	Provide name, institutional affiliation, and e-mail address of all protocol authors; provide physical mailing address of corresponding author
p16	**Contributions**	3b	Describe contributions of protocol authors and identify the guarantor of the review
n/a	**Amendments**	4	If the protocol represents an amendment of a previously completed or published protocol, identify as such and list changes; otherwise, state plan for documenting important protocol amendments
n/a	**Support**		
	**Sources**	5a	Indicate sources of financial or other support for the review
	**Sponsor**	5b	Provide name for the review funder and/or sponsor
	**Role of sponsor/ funder**	5c	Describe roles of funder(s), sponsor(s), and/or institution(s), if any, in developing the protocol
	**INTRODUCTION**		
p4-5	**Rationale**	6	Describe the rationale for the review in the context of what is already known
p6	**Objectives**	7	Provide an explicit statement of the question(s) the review will address with reference to participants, interventions, comparators, and outcomes (PICO)
	**METHODS**		
p10	**Eligibility criteria**	8	Specify the study characteristics (e.g., PICO, study design, setting, time frame) and report characteristics (e.g., years considered, language, publication status) to be used as criteria for eligibility for the review
p6-7	**Information sources**	9	Describe all intended information sources (e.g., electronic databases, contact with study authors, trial registers, or other grey literature sources) with planned dates of coverage
p8-9	**Search strategy**	10	Present draft of search strategy to be used for at least one electronic database, including planned limits, such that it could be repeated
	**Study records**		
p 11-12	**Data management**	11a	Describe the mechanism(s) that will be used to manage records and data throughout the review
p11-12	**Selection process**	11b	State the process that will be used for selecting studies (e.g., two independent reviewers) through each phase of the review (i.e., screening, eligibility, and inclusion in meta-analysis)
p11-12	**Data collection process**	11c	Describe planned method of extracting data from reports (e.g., piloting forms, done independently, in duplicate), any processes for obtaining and confirming data from investigators
p12	**Data items**	12	List and define all variables for which data will be sought (e.g., PICO items, funding sources), any pre-planned data assumptions and simplifications
p12	**Outcomes and prioritization**	13	List and define all outcomes for which data will be sought, including prioritization of main and additional outcomes, with rationale
p12-13	**Risk of bias in individual studies**	14	Describe anticipated methods for assessing risk of bias of individual studies, including whether this will be done at the outcome or study level, or both; state how this information will be used in data synthesis
	**Data**		
	**Synthesis** p13-15	15a	Describe criteria under which study data will be quantitatively synthesized
	p13-15	15b	If data are appropriate for quantitative synthesis, describe planned summary measures, methods of handling data, and methods of combining data from studies, including any planned exploration of consistency (e.g., I^2, Kendall's tau)
	p15	15c	Describe any proposed additional analyses (e.g., sensitivity or subgroup analyses, meta-regression)
	p15-16	15d	If quantitative synthesis is not appropriate, describe the type of summary planned

Table 3 PRISMA-P 2015 checklist: recommended items to include in a systematic review protocol[a] *(Continued)*

p15	**Meta-bias(es)**	16	Specify any planned assessment of meta-bias(es) (e.g., publication bias across studies, selective reporting within studies)
p15	**Confidence in cumulative evidence**	17	Describe how the strength of the body of evidence will be assessed (e.g., GRADE)

PRISMA-P Preferred Reporting Items for Systematic review and Meta-Analysis Protocols.
[a]It is strongly recommended that this checklist be read in conjunction with the PRISMA-P Explanation and Elaboration [30] for important clarification on the items. Amendments to a review protocol should be tracked and dated. The copyright for PRISMA-P (including checklist) is held by the PRISMA-P Group and is distributed under a Creative Commons Attribution License 4.0.

endorsement of PRISMA-P 2015 by journals (and potentially by other organizations) influences the completeness of reported protocols. Such an evaluation will be planned after allowing sufficient time for the wide dissemination of PRISMA-P 2015.

Implementation

The current system of implementing reporting guidelines is not optimal. At present, their primary mechanism of uptake is through endorsement by journals at their discretion, if at all. In journals that do endorse

Table 4 Proposed stakeholders, actions, and potential benefits for supporting adherence to PRISMA-P

Stakeholder	Proposed action	Potential benefits
**Funders**	Promote or mandate adherence to PRISMA-P or use PRISMA-P as a template for systematic review proposals for grant applications	Improved quality, completeness, and consistency of systematic review proposal submissions
		Standardized protocol content will improve peer review efficiency and investigator understanding of requirements
**Systematic review authors/ groups/organizations**	Use/adhere to PRISMA-P during protocol development	Improved quality, completeness, and consistency of protocol content
		Enables reviewers to anticipate and avoid future changes to review methods (i.e., outcomes)
		Increased awareness of minimum content for protocol reporting
		Improved completeness of reporting of completed reviews
**PROSPERO (and other review registries)**	Encourage the development of PRISMA-P-based protocols	Improved quality of registry entries
		Improved consistency across registry entries, protocols, and systematic reviews
**Practice guideline developers**	Use PRISMA-P to gauge the completeness of protocols and facilitate detection of selective reporting when considering reviews for guideline inclusion	Enables easy comparison across protocols, registry entries, and completed systematic reviews
**Policymakers**	Advocate use of PRISMA-P by those funding and carrying out systematic reviews	May yield better quality, more complete, and more consistent reviews to inform decision-making
**Journal editors**	Encourage compliance to PRISMA-P for authors submitting protocols for publication	Improved quality, completeness, and consistency of protocols over those published in journals not endorsing PRISMA-P
	Offer PRISMA-P as a template to assist in protocol writing for publication	Increased efficiency in protocol peer and author understanding of journal requirements
		Improved transparency and interpretation of reviews by readers
**Educators**	Use PRISMA-P as a training tool	Simplified teaching and grading of protocols
	Encourage adherence in students submitting protocols for coursework	Improved quality, completeness, and consistency of protocol content
**Students**	Develop protocols for coursework or research using PRISMA-P	Improved understanding of the minimum protocol content
		Well-trained systematic reviewer going into the workforce
