# Supplementary material for: Dietary sodium intake and overweight and obesity in children and adults: a protocol for a systematic review and meta-analysis
Source: Syst Rev. 2016 Jan 18;5:7. doi: 10.1186/s13643-015-0175-3 (PMC4717573; doi:10.1186/s13643-015-0175-3)
Supplement: Additional file 2: — Modified Newcastle–Ottawa Quality Assessment Scale. (PDF 272 kb) [file 13643_2015_175_MOESM2_ESM.pdf]

Modified Newcastle – Ottawa Quality Assessment Scale

Prospective Cohort Studies- Outcome adiposity measure

Note: A study can be awarded a maximum of one star for item number 1 and two stars for item number 2 within the Selection category, a maximum of one star for each numbered item within the Outcome category and a maximum of two stars can be given for Comparability. (Total - maximum of 7 stars)

Selection (maximum of 3 stars)

1) Representativeness of the exposed cohort

- a) truly representative of the source population ☆
- b) somewhat representative of the source population ☆
- c) selected group of users e.g. nurses, volunteers
- d) no description of the derivation of the cohort

2) Assessment of the exposure (dietary sodium/salt intake)

- a) 24-hr urine collection (1 or more) ☆ ☆
- b) 24-hr dietary recall method (1 or more) ☆
- c) Weighed dietary record (1 or more days)☆
- d) Urine sample: spot, timed or overnight
- e) Food frequency questionnaire

Comparability (maximum of 2 stars)

1) Comparability of cohorts on the basis of the design or analysis

- a) study controls for age and gender ☆
- b) study controls for energy intake ☆
- c) Only unadjusted model presented

Outcome (maximum of 2 stars)

1) Assessment of outcome

- a) objectively measured adiposity outcome (e.g. body weight and height for BMI) ☆
- b) self report body weight and height
- c) no description

2) Adequacy of follow up of cohorts

- a) complete follow up - all subjects accounted for ☆
- b) subjects lost to follow up unlikely to introduce bias - –(> 75% follow up, or description provided of those lost) ☆
- c) follow up rate <75%and no description of those lost
- d) no statement

Modified Newcastle – Ottawa Quality Assessment Scale

Cross-sectional Studies- Outcome adiposity measure

Note: A study can be awarded a maximum of one star for item number 1 and two stars for item number 2 within the Selection category, a maximum of one star for each numbered item within the Outcome category and a maximum of two stars can be given for Comparability. (Total - maximum of 7 stars)

Selection (Maximum 3 stars)

1) Representativeness of the exposed cohort (maximum of one star)

- a) truly representative of the source population ☆
- b) somewhat representative of the source population ☆
- c) selected group of users eg nurses, volunteers
- d) no description of the derivation of the cohort

2) Assessment of the exposure (dietary sodium/salt intake) (maximum of two stars)

- a) 24-hr urine collection (1 or more) ☆ ☆
- b) 24-hr dietary recall method (1 or more) ☆
- c) Weighed dietary record (1 or more days)☆
- d) Urine sample: spot, timed or overnight
- e) Food frequency questionnaire

Comparability (Maximum of 2 stars)

1) Methods to control confounding

- a) study controls for age and sex☆
- b) study controls for energy intake ☆

Outcome (Maximum of 2 stars)

1) Assessment of outcome (maximum of one star)

- a) objectively measured adiposity outcome (e.g. body weight and height for BMI) ☆
- b) self report body weight and height
- c) no description

2) Non-Response rate (maximum of one star)

- a) Non-response rate ≤20% ☆
- b) Non-response rate >20%
- c) no description

Modified Newcastle – Ottawa Quality Assessment Scale

Prospective Cohort Studies – Outcome SSB Intake

Note: A study can be awarded a maximum of one star for item number 1 and 2 stars for item number 2within the Selection category, a maximum of one star for each numbered item within the Outcome category and a maximum of one star can be given for Comparability. (Total - maximum of 6 stars)

Selection (maximum of 3 stars)

1) Representativeness of the exposed cohort

a) truly representative of the source population ✰
b) somewhat representative of the source population ✰
c) selected group of users e.g. nurses, volunteers
d) no description of the derivation of the cohort

2) Assessment of the exposure (dietary sodium/salt intake)

a) 24-hr urine collection (1 or more) ✰ ✰
b) 24-hr dietary recall method (1 or more) ✰
c) Weighed dietary record (1 or more days)✰
d) Urine sample: spot, timed or overnight
e) Food frequency questionnaire

Comparability (maximum of 1 stars)

1) Comparability of cohorts on the basis of the design or analysis

a) study controls for age and gender ✰
b) Only unadjusted model presented

Outcome (maximum of 2 stars)

1) Assessment of outcome

a) validated dietary assessment tool used to quantify SSB intake✰
b) unvalidated dietary assessment tool used to determine SSB intake
d) no description

2) Adequacy of follow up of cohorts

a) complete follow up - all subjects accounted for ✰
b) subjects lost to follow up unlikely to introduce bias - –(> 75% follow up, or description provided of those lost) ✰
c) follow up rate <75%and no description of those lost
d) no statement

Modified Newcastle – Ottawa Quality Assessment Scale

Cross-sectional Studies – Outcome SSB Intake

Note: A study can be awarded a maximum of one star for item number 1 and 2 stars for item number 2 within the Selection category, a maximum of one star for each numbered item within the Outcome category and a maximum of one star can be given for Comparability. (Total - maximum of 6 stars)

Selection (Maximum 3 stars)

<u>1) Representativeness of the exposed cohort</u>

a) truly representative of the source population ☆
b) somewhat representative of the source population ☆
c) selected group of users eg nurses, volunteers
d) no description of the derivation of the cohort

<u>2) Assessment of the exposure (dietary sodium/salt intake)</u>

a) 24-hr urine collection (1 or more) ☆ ☆
b) 24-hr dietary recall method (1 or more) ☆
c) Weighed dietary record (1 or more days)☆
d) Urine sample: spot, timed or overnight
e) Food frequency questionnaire

Comparability (Maximum of 1 stars)

<u>1) Methods to control confounding</u>

a) study controls for age and sex☆
b) Only unadjusted model presented

Outcome (Maximum of 2 stars)

<u>1) Assessment of outcome</u>

a) validated dietary assessment tool used to quantify SSB intake ☆
b) unvalidated dietary assessment tool used to quantify SSB intake
c) no description

<u>2) Non-Response rate</u>

a) Non-response rate ≤20% ☆
b) Non-response rate >20%
c) no description
